# Paradoxes in Acupuncture Research: Strategies for Moving Forward

**DOI:** 10.1155/2011/180805

**Published:** 2010-10-11

**Authors:** Helene M. Langevin, Peter M. Wayne, Hugh MacPherson, Rosa Schnyer, Ryan M. Milley, Vitaly Napadow, Lixing Lao, Jongbae Park, Richard E. Harris, Misha Cohen, Karen J. Sherman, Aviad Haramati, Richard Hammerschlag

**Affiliations:** ^1^Department of Neurology, University of Vermont, Burlington, VT 05405, USA; ^2^Division for Research and Education in Complementary and Integrative Medical Therapies, Harvard Medical School, Boston, MA 02215-3326, USA; ^3^Department of Health Sciences, University of York, Heslington, York YO10 5DD, UK; ^4^College of Pharmacy, University of Texas, Austin, TX 78712-0127, USA; ^5^Department of Research, Oregon College of Oriental Medicine, Portland, OR 97216-2859, USA; ^6^Martinos Center for Biomedical Imaging, Massachusetts General Hospital, Charlestown, MA 02129-2020, USA; ^7^Center for Integrative Medicine, University of Maryland School of Medicine, Baltimore, MD 21207-6697, USA; ^8^Physical Medicine and Rehabilitation, University of North Carolina, Chapel Hill, NC 27599-7200, USA; ^9^Department of Anesthesiology, University of Michigan, Ann Arbor, MI 48106, USA; ^10^Chicken Soup Chinese Medicine, San Francisco, CA 94103-2961, USA; ^11^Center for Health Studies, Group Health Cooperative, Seattle, WA 98101-1448, USA; ^12^Department of Physiology and Biophysics, Georgetown University Medical Center, Washington, DC 20057-1460, USA

## Abstract

In November 2007, the Society for Acupuncture Research (SAR) held an international symposium to mark the 10th anniversary of the 1997 NIH Consensus Development Conference on Acupuncture. The symposium presentations revealed the considerable maturation of the field of acupuncture research, yet two provocative paradoxes emerged. First, a number of well-designed clinical trials have reported that true acupuncture is superior to usual care, but does not significantly outperform sham acupuncture, findings apparently at odds with traditional theories regarding acupuncture point specificity. Second, although many studies using animal and human experimental models have reported physiological effects that vary as a function of needling parameters (e.g., mode of stimulation) the extent to which these parameters influence therapeutic outcomes in clinical trials is unclear. This White Paper, collaboratively written by the SAR Board of Directors, identifies gaps in knowledge underlying the paradoxes and proposes strategies for their resolution through translational research. We recommend that acupuncture treatments should be studied (1) “top down” as multi-component “whole-system” interventions and (2) “bottom up” as mechanistic studies that focus on understanding how individual treatment components interact and translate into clinical and physiological outcomes. Such a strategy, incorporating considerations of efficacy, effectiveness and qualitative measures, will strengthen the evidence base for such complex interventions as acupuncture.

## 1. Introduction

The 1997 National Institutes of Health (NIH) Consensus Development Conference on Acupuncture was a landmark event in the history of acupuncture research [1]. The Consensus Statement concluded, “There is sufficient evidence of acupuncture's value to expand its use into conventional medicine and to encourage further studies of its physiological and clinical value.” It also emphasized that conclusions regarding acupuncture's efficacy and safety for most conditions were significantly limited by the low number of methodologically sound research trials. 

In November 2007, the Society for Acupuncture Research (SAR) held an international symposium aimed at presenting and discussing the progress made in acupuncture research during the decade following the NIH Consensus Development Conference. The symposium presentations, as well as their published summaries [2–4], unequivocally showed that the field of acupuncture research has significantly expanded and matured since 1997. Phase II/III sham-controlled clinical trials have been successfully completed, and a broad range of basic research studies have identified numerous biochemical and physiological correlates of acupuncture (see Box 1 for overviews of conclusions from the summaries of clinical and basic research presented at the 2007 symposium). However, intriguing paradoxes emerged during the symposium and the writing of the summaries. Proceedings from our 2007 conferences as well as syntheses of our findings from systematic reviews were discussed at a structured SAR Board workshop in October, 2008 (held at Georgetown University and facilitated by Aviad Haramati, a non-SAR member). The workshop resulted in the articulation of two primary paradoxes as well as an outline for this White Paper. Drafts of this paper were then written by a subset of the authors, with eventual input from all Board members and a series of external reviewers. 

The emergent potential paradoxes that frame this paper are the

1. A large number of well-designed clinical trials have reported that true acupuncture is superior to usual care, but does not significantly outperform sham acupuncture, findings apparently at odds with traditional theories regarding acupuncture point specificity and needling technique.
2. While many studies in animal and human experimental models have reported physiological effects that vary as a function of needling parameters (e.g., needle insertion depth, mode of stimulation), the extent to which these parameters influence therapeutic outcomes in clinical trials is unclear.

The goal of this White Paper is not to present a detailed review of the literature. Rather, this paper aims to identify gaps in current knowledge that underlie these paradoxes and to recommend research strategies that can help deconstruct and resolve them. We propose that resolution of the paradoxes lies in applying a bidirectional translational research approach, in which clinical trial design is informed by knowledge of mechanisms underlying acupuncture and by results from pragmatic trials of “real world” clinical practice. With this approach, findings from clinical trials will better inform the role of acupuncture in our evolving health care system.

## 2. Key Acupuncture-Related Definitions

The field of acupuncture research includes a number of terms that have created confusion and impeded progress, the word “acupuncture” notwithstanding. The term “acupuncture” has been used in the literature to convey not only the simple process of inserting a needle but also to describe a more complex intervention that includes a traditional exam, diagnosis, and the incorporation of additional modalities such as massage and moxibustion. “Sham acupuncture” and “acupuncture points” are other examples of terms fraught with confusion in the literature (*see Box 2 for definitions of four key terms and concepts that we rely on in this paper*).

## 3. Gaps in Knowledge Underlying Paradox 1

The simplest explanation for Paradox 1 is that, when no differences are found between verum and sham acupuncture treatments, the effects of both are due to nonspecific factors (e.g., expectancy, contact time, device-related ritual) rather than needling or other components specific to acupuncture. If this is the case, the implication would be that the mechanisms underlying the therapeutic effects of acupuncture are essentially placebo-related. A central thrust of this White Paper is that there are other explanations that are just as plausible since a number of factors that could lead to Type II errors (i.e., false negatives) remain poorly understood: (1) both verum and sham needling may have therapeutic effects, (2) acupuncture-specific nonneedle and/or needle components may be retained in sham treatments, and/or (3) the therapeutic effects of the provision of verum acupuncture treatments in the context of clinical trials may be suboptimal.

### 3.1. Factors Potentially Contributing to “Therapeutic” Effects of Sham Acupuncture Treatments

A major factor potentially underlying Paradox 1 is the lack of consensus on which component(s) of acupuncture needling are therapeutically important. There has been little systematic investigation regarding the importance of needle placement and depth, type and intensity of stimulation, needle number, and so forth [5], as well as the mechanisms by which these parameters influence immediate and longer-term physiological responses. Until research clarifies these issues, it will be difficult to interpret the effects of sham needling and the extent to which such approaches can be used as valid controls in clinical trials. Rational development of a placebo in pharmaceutical trials is based on knowledge of how the test drug is absorbed, metabolized, and recognized by molecular receptors, initiates biochemical and/or physiological events, and is inactivated. The challenge in designing appropriate sham acupuncture is that ignorance of acupuncture mechanism prevents us from knowing what to avoid when inserting a sham acupuncture needle [6]. In particular when numerous sham needles are inserted, there may be a cumulative, beneficial effect resulting from multiple stimulations of superficial sensory nerve endings or connective tissue (two commonly discussed targets of acupuncture needling). 

Sham acupuncture treatments have consistently been shown to have greater therapeutic effects than conventional placebos [7–10]. Placebo mechanisms include a complex spectrum of phenomena influenced by emotions as well as psychosocial and sensory cues [11]. It is thus plausible that factors specific to acupuncture (needling and/or nonneedling) may enhance placebo responses during verum and sham acupuncture treatments [12]. An interesting possibility is that the heightened focus on specific body locations caused by (true or sham) needle insertion may amplify local analgesic responses such as those demonstrated with spatially directed placebos [11, 12]. 

An additional factor that may contribute to the therapeutic effect of sham acupuncture treatments is the strong practitioner-patient interactions that commonly take place during interventions [13]. One specific component of these interactions is practitioners' *intention* for a therapeutic outcome, traditionally described as *yi*. Some research has begun to experimentally investigate the therapeutic effects of healing intention as well as possible mechanisms associated with these effects [14, 15]. 

**Figure 1:**
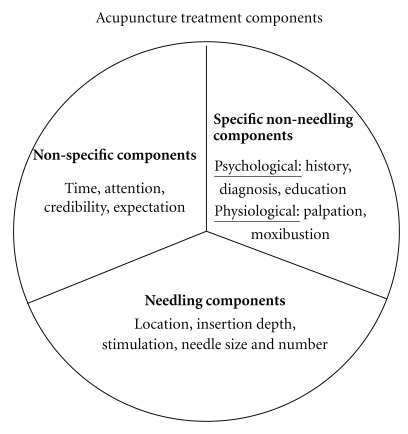
Components of acupuncture treatments broken down into nonspecific versus specific nonneedling versus needling. Specific components refer to aspects of acupuncture treatments that are characteristic of traditional acupuncture practice, as opposed to nonspecific generic components that are present in other types of treatments (See Box 2).

As Figure 1 indicates, acupuncture treatments are complex, multicomponent interventions. In sham-controlled trials that attempt to control only for needling, several other potentially therapeutic acupuncture-specific components may be present in the control group. This would be the case for certain diagnostic processes, such as palpation for sites of chronic pain. The resultant increase in the therapeutic effectiveness of the sham arm would decrease the effect size observed in the study. Recent research suggests that the magnitude of nonneedling specific effects provided in the context of acupuncture therapy may be substantial [16], but systematic research has yet to identify the extent and mechanisms through which therapeutic effects occur.

### 3.2. Factors Potentially Contributing to Decreased Therapeutic Effects of Verum Acupuncture Treatments in Clinical Trials

In contrast to drug trials that evaluate well-defined and consistent pharmaceutical agents, acupuncture treatment protocols in clinical trials are rarely validated using objective, standardized approaches. Moreover, studies attempting to develop valid protocols based on consensus of multiple acupuncturists have revealed significant pluralism regarding what constitutes appropriate treatment [17]. Unlike comparative drug trials, few rigorous studies have compared the relative efficacy of different acupuncture treatment components within protocols (e.g., number and choice of acupuncture points, type and duration of stimulation). In an attempt to create repeatable acupuncture protocols for clinical trials, many studies simplify and/or modify clinical practice (e.g., limit individualization of treatments, restrict use of co-interventions such as moxibustion, and impose restrictive standard operating procedures, such as limited patient-practitioner interaction). When only a limited number of components are evaluated in a clinical trial, these components are often chosen without a clear rationale and may not be the most therapeutically active. Consequently, the potential synergy between multiple components may be lost, resulting in a decrease in effect size [2, 6, 18]. Within any of these examples, effect sizes may be small due to use of suboptimal verum treatment protocols. 

In summary, possible explanations for the generally small effect sizes observed in many sham-controlled trials are the following

- Verum and sham treatments are equivalent, because the therapeutic effects of both are due to nonspecific effects (e.g., expectancy, generic psychosocial interactions).
- Specific therapeutic effects of acupuncture treatments do exist but are not detected in sham-controlled trials because
  - sham acupuncture treatments are not inert (i.e., sham needling and/or nonneedling components of sham treatments have therapeutic effects), and/or
  - verum acupuncture treatments are less than optimal.

## 4. Gaps in Knowledge Underlying Paradox 2

As with Paradox 1, a possible explanation for Paradox 2 is that the needle-related physiological effects observed in basic science experiments are unrelated to therapeutic effects. Another possible explanation is that at least some of the physiological effects of needling are therapeutic, but these effects are difficult to demonstrate in the context of a clinical trial.

### 4.1. Results of Basic Research May Not Be Directly Applicable to Clinical Research

As in most medical research, experiments in healthy humans and animal models have limitations in their applicability to clinical research. To date, the large majority of basic science research on acupuncture needling has been performed in healthy humans and animals; it is likely that physiological responses to acupuncture needling are different in pathological states [19]. In particular, effects of acupuncture needling gleaned from *acute* pain models (animal or human) may have limited value for informing clinical trial design and practice-based treatment of *chronic* pain conditions given the emerging view of chronic pain as a qualitatively distinct neurological dysfunction from acute pain [20]. Further, while some physiological responses to acupuncture needling have been related to clinically relevant responses in animal models (e.g., intestine motility) [21], it is less clear to what degree these responses are related to therapeutic outcomes in human subjects with specific diseases (e.g., IBS). Moreover, electrical stimulation of acupuncture needles tends to be used more frequently in basic science experiments compared with clinical trials, which could account for some of the discrepancies between basic and clinical studies. It is evident that much remains to be learned about which physiological effects of acupuncture needling are clinically relevant. 

Mechanistic studies of acupuncture treatments and needling are also seriously hampered by three additional factors. First, similar to biomedical research, a significant proportion of acupuncture research evaluates conditions for which the pathophysiology is largely unknown, therapeutic outcomes are mostly subjective, and no good animal models or biomarkers exist (e.g., chronic low back pain). Second, there are no current animal models that investigate the mechanisms of acupuncture treatments within the explanatory framework of traditional East Asian medicine. Third, we do not understand what, if any, physiological equivalents exist in animals *or* humans for functional concepts that are key to the traditional rationale underlying acupuncture treatments, such as acupuncture points, meridians, and “qi”.

### 4.2. Lack of Understanding of Why Effect Sizes Appear Larger in Physiological Experiments than in Clinical Trials

As discussed above, clinically relevant physiological effects (e.g., anti-inflammatory or intestinal motility effects) demonstrated in experimental models may be masked in clinical trials by the magnitude and variability of the multiple components of verum acupuncture treatment. In a sham-controlled clinical trial for chronic pain, an anti-inflammatory effect of verum needling may indeed be present, but the magnitude of this effect may be proportionally small compared with large and variable therapeutic effects produced by both verum and sham nonneedling components (e.g., interaction with practitioner). A potentially important difference between basic science studies and clinical trials is that the former tend to use small numbers of needles, inserted at the same locations in every animal or human subject, while the latter, for the sake of reflecting clinical practice, often use 10 to 20 needles per session with at least some degree of practitioner-permitted choice of needling location. Thus, a small but specific therapeutic “signal” due to needling may be detectable in a physiological experiment but not readily detectable in clinical trials due to the additional “noise” in the system. 

In summary, possible explanations for why specific effects of needling are clearly demonstrable in basic science studies, but not as evident in sham-controlled clinical trials are as follows:

- Some or all of the needle-related physiological effects observed in basic science experiments are unrelated to therapeutic effects.
- Acupuncture needling does have therapeutic effects, but these effects are proportionately small compared with nonneedle and nonspecific effects in clinical trials.
- Sham-controlled clinical trials are compromised methodologically because
  - the influence of needling parameters for verum and sham needling are poorly understood, and
  - objective biomarkers relevant to the commonly treated clinical conditions are lacking.

## 5. Research Needed to Resolve the Paradoxes: Recommendations for the Next Decade

The 2007 SAR International Symposium confirmed a growing sense of puzzlement in the acupuncture research community regarding the apparent disparities between the results of basic science and sham-controlled trials on the one hand and the clinical practice of acupuncture on the other. A number of interrelated challenges must be met to resolve the paradoxes identified in this paper. At the outset is the need to resolve the ambiguity of the term “acupuncture” within the scientific literature. To this end, we encourage the scientific community to adopt the terms “acupuncture *needling*” and “acupuncture *treatment*” (as defined in Box 2) and to maintain rigorous clarity when describing the methods used in the publications of basic science and clinical studies. 

 We also encourage the scientific community to adopt an overarching strategy that includes clinical practice-informed approaches on the one hand and basic science approaches on the other, both of which should be aimed at better informing the design of the acupuncture treatment and sham controls in clinical trials. As examples, “whole systems research” [22], pragmatic trials [23] and surveys of real-world clinical practice can help to provide a more comprehensive understanding of the multiple components that potentially underlie the therapeutic effects of acupuncture treatment. 

Basic research, for its part, can help to define additional biomarkers that may be of importance in distinguishing between effects of real and sham acupuncture treatments. Importantly, basic research studies should focus mainly on human and animal models of chronic disease, in addition to healthy human volunteers and acute animal models, to better ensure that findings will be relevant to the design of clinical trials.

### 5.1. Clinical Practice-Informed Approaches: Understanding Acupuncture Treatments as Complex Interventions

 One possible contributing factor to Paradox 1 is that important components of real treatments are inadvertently included in the sham treatment protocol due to an insufficient understanding of the complexity of acupuncture treatment. Simply stated, an appropriate sham procedure cannot be designed without sufficient knowledge of what needs to be “shammed”. Ideally, a sham procedure that controls for nonspecific components of acupuncture treatments would include none of the specific needling and nonneedling components but would still be credible. A potentially valuable approach is to creatively adapt the methodology of evaluating complex interventions [24, 25] to the study of acupuncture treatments. The goal here is not to reduce a complex system of care to an exhaustive list of individual components and to try to study how each and every component contributes to a therapeutic effect, which would be an impossible task. Rather, a more practical and realistic goal is to use clinical experience to identify key components specific to acupuncture treatments and to systematically study their therapeutic effects as well as their impact on basic physiological processes. This approach needs to start off using clearly defined control procedures designed to test the specific component that is being evaluated (i.e., needle location, electrical stimulation), rather than poorly understood sham procedures that attempt to control for too many factors. Once the effects of some of the individual components are identified, a clearer understanding will emerge of how to create a sham protocol that appropriately controls for nonspecific effects of acupuncture by mimicking but not contributing to the specific acupuncture-related therapeutic process.

In seeking to evaluate how the parameters of acupuncture needling contribute to acupuncture treatment, it is also of major interest to learn what is most important to practitioners and patients among the multiple components of acupuncture beyond simply needling. This can be done by using tools of qualitative research to record the actual experience of practitioners and patients [26, 27] and may be supplemented by using emerging medical imaging and biomarker approaches to evaluate the physiological activity of practitioners and patients during the therapeutic encounter [28]. The complexity of acupuncture interventions makes it unlikely that even a battery of standardized outcomes will adequately capture the richness of practitioners' experiences, which may inform optimal study design. Qualitative methods can be used to explore the meaning that patients ascribe to an intervention, the process and context by which healing occurs, outcomes that are relevant and meaningful to patients, and how interventions fit within everyday lives [29]. Recent CAM studies that have combined qualitative and quantitative methods have demonstrated that this integrated research approach can be very informative [27, 29, 30]. A parallel, valuable research design, as mentioned above, is to compare whole systems of CAM and biomedical interventions, each delivered in a manner that best reflects real-world practice [22]. Once effectiveness of the whole system is demonstrated, future trials can seek to identify the most effective balance of the system's component modalities. 

Traditional explanations of acupuncture treatment include emphasis on numerous intricacies stemming from the concept of treatment individualization. For example, in the traditional Chinese medicine (TCM) style of acupuncture, “pattern differentiation” is purported to be paramount for effective treatments and influences choice of needle location, needle stimulation techniques, and treatment frequency and duration. Consensus among acupuncturists regarding TCM diagnosis has ranged from good to poor in various interrater reliability studies [31–33] but was found to markedly improve with training using a questionnaire [34]. In a condition such as low back pain, systematic study could reveal an optimal treatment protocol for a specific patient population based on both biomedical and traditional diagnostic techniques. This protocol could then be used to determine which of the traditional concepts are most important to optimal treatment. Outcomes should be subjective as well as objective and attempt to correlate clinical effectiveness with relevant biomarkers.

### 5.2. Basic Science-Informed Approaches: Exploring Acupuncture Point Anatomy, Physiology, and Clinically-Relevant Biomarkers

A particularly controversial question is the issue of “point specificity” with respect to clinical and/or physiological effects of acupuncture needling. It remains unclear whether effects observed at certain acupuncture points (e.g., P6 on the wrist overlying the median nerve) are due to proximity of the needle to a major nerve, rather than its location at a specific acupuncture points. Few studies, for example, have included nonacupuncture point controls located along the same nerve as the tested acupuncture point. More studies including such controls therefore are urgently needed to advance our understanding of this important issue. 

A better understanding of potential differences in physiological responses elicited at acupuncture points and nonacupuncture points also is important for the future design of sham controls in clinical trials. Development of a modern scientific rationale for the traditional theories related to insertion of needles at specific locations is particularly challenging. Although it is possible that acupuncture points are truly unique anatomical structures, so far this has not been sufficiently and rigorously corroborated by research. In particular, a recent systematic review found little evidence in support of using skin conductance or impedance to identify acupuncture points [35]. However, it is also possible that acupuncture points may represent effective—but not unique—sites at which sensory afferent stimulation produces beneficial effects or at which an underlying regulatory system might be activated. Therefore, additional systematic reviews are needed to assess the numerous claims that acupuncture points have distinct histological and/or biochemical characteristics (e.g., neurovascular bundles, nerve endings, mast cells). This will encourage rigorous attempts to replicate the most promising findings. However, if high-quality studies consistently fail to demonstrate clear criteria that can be used to identify “nonacupuncture points”, our understanding of acupuncture will remain limited and the use of such sites in sham needling procedures will remain problematic. 

Studies are needed to identify the physiological effects associated with individual components of acupuncture, both needling and nonneedling. These include more in-depth and systematic investigations regarding the importance of such parameters as needle placement and depth [36], stimulation type and intensity, and needle number as well as the mechanisms by which these parameters influence physiological responses (immediate and longer-term) and modify biomarkers [37]. Also needed are assessments of putative physiological effects of nonspecific needling components integral to acupuncture treatments [38] as well as physiological effects of nonspecific treatment components, for example, patient expectation [16]. In order to better understand why sham needling (superficial at nonacupuncture point sites) has relatively similar clinical effects as true needling at acupuncture points [39], experiments in humans and animals should simultaneously examine the effects of verum and sham needling (using one or multiple needles) on local tissues and the nervous system. 

Biomarkers that relate to a given pathological condition and improve with treatment are needed to objectively evaluate treatment efficacy both in humans with these conditions and in animal models. If an understanding of disease pathophysiology is lacking, patients may be studied who have similar symptoms but who may have quite different disease mechanisms. Thus, the question “is acupuncture effective for chronic low back pain?” is likely not precise enough since low back pain is heterogeneous and may include subgroups with pathology that responds to treatment and subgroups that do not [17, 40]. Moreover, developing biomarkers associated with concepts of traditional East Asian medicine would allow not only objective testing of acupuncture treatments in their “original” context, but also potentially expand our fundamental understanding of human physiology and pathophysiology.

An additional recommendation is that reliable biomarkers and clinical outcome measures of both *immediate* and *delayed *(hours to years) physiological responses to needling in humans be developed to facilitate assessments of effective needling parameters. This can be designed as a two-phase program in which the outcome measures are developed and correlated first in healthy volunteers, then repeated for assessment of clinical validity in patients. For example, quantification of changes in pulses (informed by Chinese and/or Japanese acupuncture theory) could not only qualify as an immediate response measure but would have an added advantage of beginning to develop biomarkers from within the acupuncture explanatory model. Further, suggestions as to why some parameters of needle stimulation appear to have physiological effects in basic science studies but are not associated with specific therapeutic effects in clinical trials (Paradox 2) may emerge from assessing immediate and/or delayed responses to needling (e.g., brain activation patterns) of healthy volunteers in comparison with medically diagnosed patients using the same specific needling parameters, for example, needle insertion depths, mode and amount of stimulation.

### 5.3. Designing Appropriate Control Procedures

Rational design of a sham needling procedure requires knowing “what to mimic” *and* “what to avoid”. Most sham designs (e.g., retractable needles) are based mainly on the former consideration (aspects of treatment the patient sees) with insufficient attention to the latter. Thus, development of sham controls should be based on a systematic basic science evaluation of the components of acupuncture treatments (e.g., insertion depth, needle location, needle stimulation) that the sham procedure should avoid. In order to gain clarity on the interpretation of these studies, we urge the scientific community to clearly state the hypothesis that is being tested and select a control procedure(s) that will most specifically test this hypothesis. For example, if a given physiological response in a basic science experiment is hypothesized to be due to electrical stimulation of acupuncture needles, an appropriate control would be needle insertion at the same location and depth but without electrical stimulation. If, on the other hand, the hypothesis is that there is an interaction between electrical stimulation, and needle location (i.e., electrical stimulation has different effects at acupuncture points versus nonacupuncture points), then four experimental groups are needed (acupuncture points with stimulation, acupuncture points without stimulation, nonacupuncture points with stimulation and nonacupuncture points without stimulation). Thus, the frequent practice of comparing needle stimulation at acupuncture points to no stimulation at nonacupuncture points should be interpreted with caution as it risks confounding the two variables (i.e., electrical stimulation and needle location). Similarly, the common practice of comparing acupuncture points to nonacupuncture points in a different body region (e.g., limb versus abdomen) risks confounding point type (i.e., acupuncture points versus nonacupuncture points) and body region unless additional controls are included. 

We thus encourage future clinical trials including both usual care (to test the effectiveness of acupuncture) as well as appropriate control group(s) designed to test the efficacy of specific treatment components (e.g., control needling at the same location and depth but without manual or electrical stimulation). Such trials would be further strengthened by the inclusion of objective measurements of pathology relevant to the condition being treated. A summary of combined recommendations based on the paradoxes identified in this paper is shown in Table 1. 

## 6. Conclusions

The experience gained by acupuncture researchers over the last ten years is invaluable both for the field of acupuncture itself and as an example for the study of other complex treatments that may wrestle (now or later) with similar issues. It is therefore vital to recognize the lack of clear difference between verum and sham acupuncture treatments in clinical trials, as well as the documented physiological effects of acupuncture needling in basic science studies. It is now the important responsibility of acupuncture researchers to face these results squarely and move the field forward. 

It is conceivable, as stated above, that the therapeutic effects of acupuncture treatments are all or largely based on nonspecific, placebo-related responses. If this is true, the implications for the investigation and practice of acupuncture are profound as this would certainly raise interesting questions as to the value of specific acupuncture point location and needling techniques. Before we consider such a perspective, however, it is important that the issues outlined in this White Paper are addressed.

An opinion commonly voiced in the acupuncture community is that “acupuncture cannot be studied using randomized controlled trials”. We strongly disagree. Rather, we encourage clinicians and scientists to recognize that much patience and hard work will be needed before we fully understand how best to use the tools at our disposal, as well as develop new ones, to study this complex form of treatment. As has been shown in the field of ecology, complex phenomena are best tackled using a pluralistic strategy including both “top-down” approaches that evaluate the behavior of a whole “intact” system, as well as “bottom-up” approaches that evaluate smaller-scale cause-and-effect links. Acupuncture treatments thus should be studied both from the “top down” as multicomponent “whole-system” interventions employing a pragmatic systems perspective, as well from the “bottom up” as mechanistic studies that focus on understanding individual treatment components and how the effects of these components interact and translate into clinical outcomes. 

**Figure 2:**
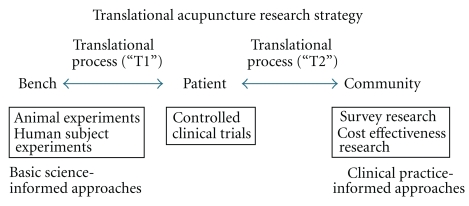
Illustration of bidirectional translational acupuncture research schema.

Viewed through the lens of translational research (Figure 2), the schema that we propose for the resolution of Paradox 1 is that basic research must inform clinical research (on how best to design an appropriate sham acupuncture procedure) so that clinical research can better inform clinical practice as well as health care policy and reimbursement decisions. For Paradox 2, the translational schema is that clinical practice should inform basic and clinical research regarding the development of clinically appropriate outcome measures and biomarkers as well as the evaluation of appropriate verum and sham treatments in efficacy trials. 

Creation of an acupuncture research agenda involves more than formulating lists of individualized goals for basic science and clinic research. In addition to providing a framework for addressing the paradoxes, the translational research approach will deepen our understanding of complex systems of care and refine our perspectives of human health and disease. Further, in calling for bidirectional translational research, SAR emphasizes the importance and value of building clinical relevance into the design of clinical as well as basic science research. The idea that research design should be framed in the context of ecological validity is an imperative, especially in light of the limited funds available for acupuncture research and its primary raison d'être, which is improved patient care. A bidirectional translational research strategy, incorporating considerations of efficacy, effectiveness, and qualitative measures, will result in a broader and stronger basis of support for the study of complex treatments in general, including acupuncture and other CAM interventions.

## Figures and Tables

**Box 1:**
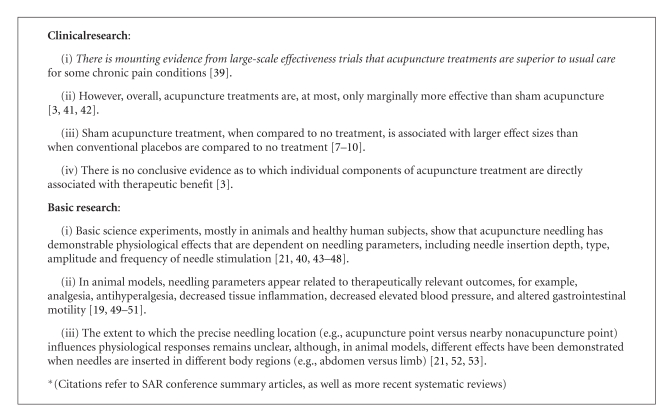
Summary of conclusions emerging from the 2007 Society for Acupuncture Research Conference* [3, 4].

**Box 2:**
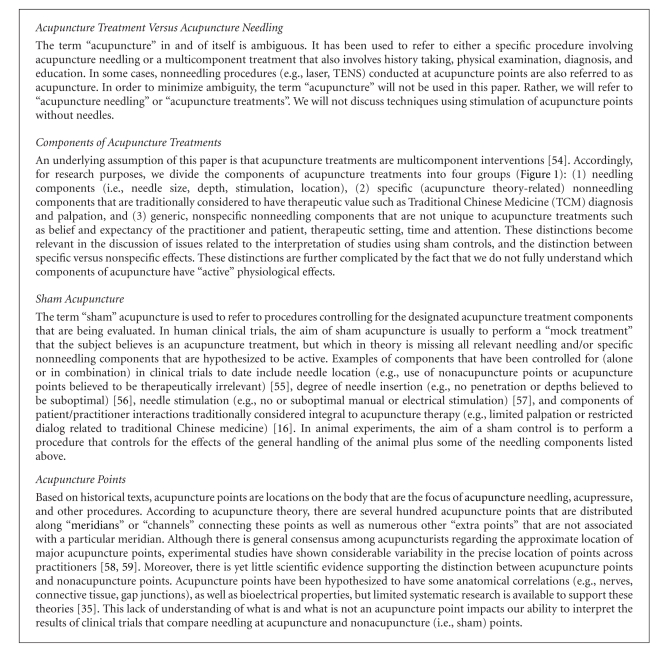
Acupuncture-related definitions.

**Table 1:** Summary of combined recommendations for future research based on the paradoxes identified in this paper.

**(a) Recommendations based on paradox 1**	**Expected benefits for future clinical trials**
Understanding the physiological effects of acupuncture needling using appropriate controls	Prevention of needle-specific physiological effects in sham treatment

Identifying key physiological and psychological *nonneedle* components of acupuncture treatment	Prevention of specific nonneedle effects in sham treatment

Recognizing the extent to which acupuncture provided in the context of a clinical trial reflects acupuncture in real world clinical practice	Increased ecological validity

**(b) Recommendations based on paradox 2**	**Expected benefits for future clinical trials**

Translation of physiological effects of acupuncture observed in *acute* animal models and healthy humans to clinical treatment of complex *chronic* conditions	Improved correlations between needling parameters, biomarker changes and clinical outcomes

Translation of basic science findings with electro-acupuncture parameters using few needles to more traditional manual needling using multiple needles	Improved understanding of complex manual needling protocols as well as electrostimulation

Cross-correlations between Traditional Chinese Medicine (TCM) and biomedicine syndromes	Use of biomarkers to TCM entry criteria as objective outcome measures
